# An analytical framework for prediction accuracy of estimated daily yields in 3-times-daily milking herds

**DOI:** 10.3168/jdsc.2026-1072

**Published:** 2026-08-17

**Authors:** Xiao-Lin Wu, George Wiggans, Ashley Ling, Malia J. Caputo, Curtis P. Van Tassell, Ransom L. Baldwin, John B. Cole, Javier Burchard, João Dürr

**Affiliations:** 1 Council on Dairy Cattle Breeding, Bowie, MD 20716; 2 Department of Animal and Dairy Sciences, University of Wisconsin, Madison, WI 53706; 3 USDA Animal Genomics and Improvement Laboratory, Beltsville, MD 20705; 4 Department of Animal Science, North Carolina State University, Raleigh, NC 27695; 5 Department of Animal Sciences, Donald Henry Barron Reproductive and Perinatal Biology Research Program, and the Genetics Institute, University of Florida, Gainesville, FL 32611; 6 Department of Animal Biosciences, University of Guelph, ON, Canada N1G 2W1

## Abstract

Accurate estimation of daily milk, fat, and protein yields from 3-times-daily (3×) milking data relies on multiplicative correction factors derived from proportional-yield modeling. Although this method has been used for more than 40 yr in the United States, the determinants of prediction accuracy and the practical limits of further improvement remain poorly understood. The objective of this study was to develop an analytical framework to quantify and decompose the sources of prediction error in estimated daily yields and to clarify how accuracy can be improved. Building on a reparameterized proportional-yield model centered on an 8-h milking interval, we derived an approximation for the expected correlation between observed and estimated daily yields. The total prediction error variance was decomposed into intrinsic stochastic variability and parameter estimation-induced uncertainty. The method was evaluated through rotational cross-validation using 3× milking data collected from 4 US Holstein herds during 2023 to 2025. Analytical accuracies closely matched empirical correlations between observed and estimated yields for milk, fat, and protein. Across herds and traits, intrinsic stochastic variability accounted for nearly all prediction error variance (e.g., >99%), whereas estimation-induced variance was negligible. These results demonstrate that attainable accuracy is fundamentally limited by biological and sampling variability in yields rather than by estimation uncertainty in regression parameters once sufficient data were available for training. Consequently, meaningful improvements in prediction accuracy will require refinement of the statistical model or the sampling procedure, for example, using a multiple-day average of records to effectively reduce intrinsic stochastic variance. The proposed analytical framework provides a practical and effective tool for diagnosing the underlying sources of prediction error and informing strategies to improve daily yield estimation in 3× milking systems.

## Graphical Abstract

**Figure:**
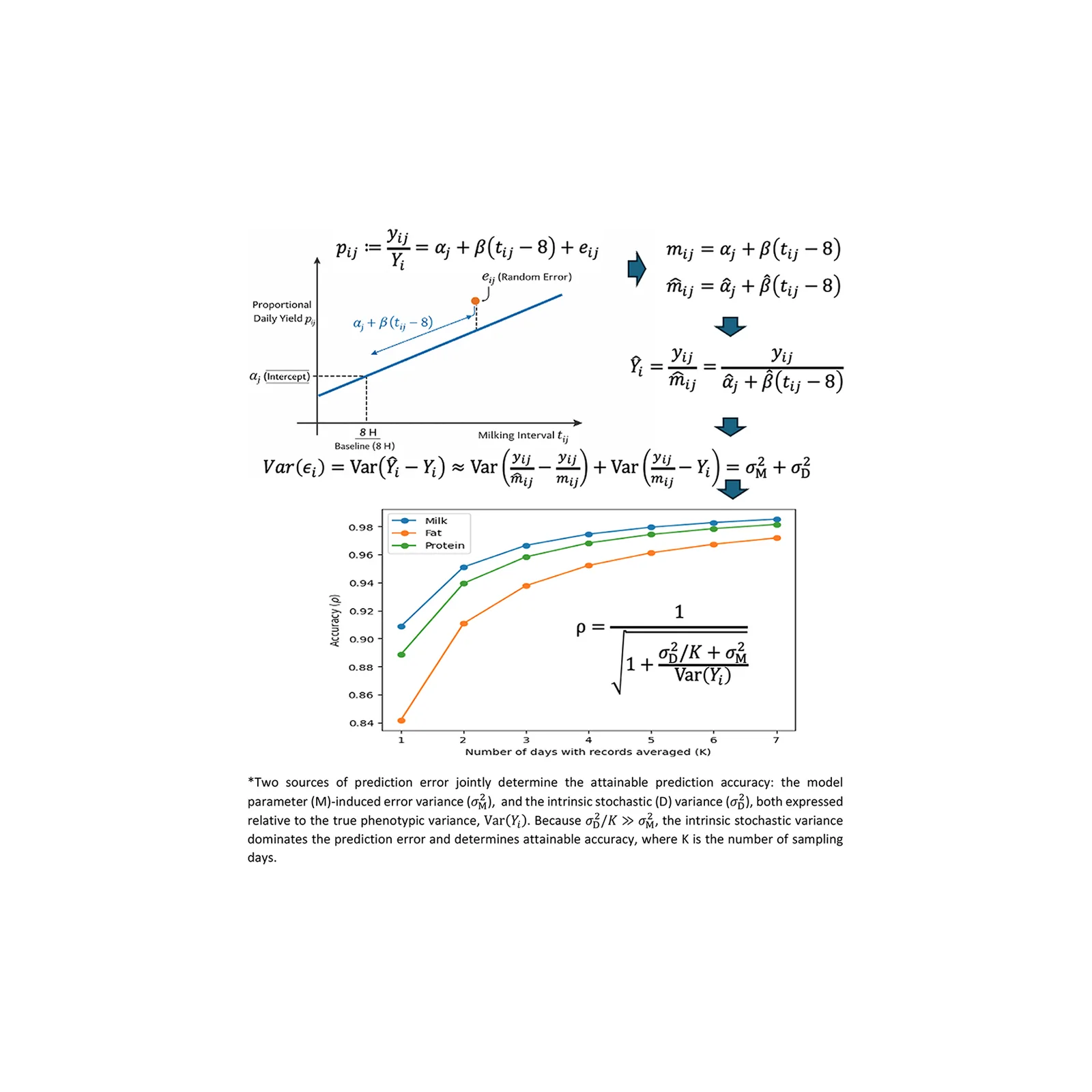

In the United States, three-times-daily (3×) milking is widely practiced, particularly in large dairy operations (USDA-APHIS, 2016). However, the DHI program typically records only 1, or occasionally 2, milkings per test day. Consequently, accurate and unbiased estimates of the total 24-h yield from these partial observations remain essential to ensure reliable recordkeeping, genetic evaluation, and herd management (ICAR, 2022). To address this need, Wiggans (1986) proposed a least-squares-based proportional-yield modeling approach to derive multiplicative correction factors in 3× daily milking herds.

Under the proportional-yield model, the parameters used to estimate daily yields from partial yields in 3× milking herds were developed more than 40 yr ago in the United States. However, no analytical framework has been developed to quantify the attainable prediction accuracy within the yield factor correction framework.

The objective of this study was to develop an analytical method to decompose the sources of prediction error for estimating daily yields and quantify their relative contributions to attainable accuracy using a reparameterized proportional-yield model (Wu et al., 2026). By decomposing the total mean-squared prediction error into intrinsic stochastic variability and parameter uncertainty, we identify the factors that determine attainable accuracy and examine the implications for improving accuracy through model refinement or an enhanced sampling design.

To improve the biological interpretation of parameters in the proportional-yield model proposed by Wiggans (1986), we recently proposed a reparameterized model that centers the milking interval at 8 h (Wu et al., 2026). Thus, the model becomes

[1] $$p_{ij}=\alpha_{j}+\beta \left(t_{ij}-8\right)+e_{ij},$$

where *p_ij_* = *y_ij_*/*Y_i_* is the proportional yield for the *j*th milking observed on the *i*th cow, *t_ij_* is the milking interval (h), and *e_ij_* has mean zero and variance $\sigma_{e}^{2}.$

Denote the deterministic component of [1] as

[2] $$m_{ij}=\alpha_{j}+\beta \left(t_{ij}-8\right)$$

and its estimator

[3] $${\hat{m}}_{ij}={\hat{\alpha }}_{j}+\hat{\beta }\left(t_{ij}-8\right).$$

Using 1/1m^ijm^ij as the multiplicative correction factor (Wiggans, 1986; Wu et al., 2023b), which depends solely on the milking interval length, the estimated daily yield is

[4] $${\hat{Y}}_{i}=\frac{y_{ij}}{{\hat{m}}_{ij}}=\frac{y_{ij}}{{\hat{\alpha }}_{j}+\hat{\beta }\left(t_{ij}-8\right)}.$$

Subtracting the true daily yield from the prediction gives the prediction error

$$<!--ERROR-14INMATHTYPECONVERSION.PLEASECORRECTMANUALLY-->\text{\textbackslash{}[}{\;}_{i}:={\hat{Y}}_{i}-Y_{i}=\frac{y_{ij}}{{\hat{m}}_{ij}}-Y_{i}=\left(\frac{y_{ij}}{{\hat{m}}_{ij}}-\frac{y_{ij}}{m_{ij}}\right)+\left(\frac{y_{ij}}{m_{ij}}-Y_{i}\right).\text{\textbackslash{}]}$$

Here, the first term on the right-hand side represents the model parameter (M)-induced prediction error arising from parameter estimation uncertainty in the yield correction factors, whereas the second term represents the intrinsic stochastic (D) component of prediction error arising from using the conditional mean *m_ij_* in place of the stochastic proportional yield *p_ij_*.

Assuming independence between training and testing data so that the 2 sources of prediction error are uncorrelated, the variance of the total prediction error is approximated by

[5] $$<!--ERROR-14INMATHTYPECONVERSION.PLEASECORRECTMANUALLY-->\$Var\left({\;}_{i}\right)=Var\left({\hat{Y}}_{i}-Y_{i}\right)\approx \sigma_{M}^{2}+\sigma_{D}^{2},\$$$

where $\sigma_{M}^{2}=Var\left(\frac{y_{ij}}{{\hat{m}}_{ij}}-\frac{y_{ij}}{m_{ij}}\right)$ and $\sigma_{D}^{2}=Var\left(\frac{y_{ij}}{m_{ij}}-Y_{i}\right).$

The M-source prediction error variance $\left(\sigma_{M}^{2}\right)$ arises from imperfect knowledge of model parameters (α, β) and the use of a finite training sample. These sources of uncertainty propagate into the estimated daily yield through the estimated yield correction factors. When ${\hat{m}}_{ij}=m_{ij},$ we have $\sigma_{M}^{2}=0.$

In contrast, the D-source prediction error variance $\left(\sigma_{D}^{2}\right)$ persists even when the true model parameters are known under the proportional-yield model. This component arises from replacing the stochastic proportional yield *p_ij_* with its conditional mean *m_ij_*. Because the true daily yield satisfies Yi=yij/yij(mij+eij)(mij+eij), this term reflects the effect of the residual variation *e_ij_*, which enters nonlinearly through the denominator and cannot be recovered from a single observation. However, even if ${\hat{m}}_{ij}$ perfectly estimates *m_ij_*, the intrinsic prediction error does not vanish because $Y_{i}=\frac{y_{ij}}{p_{ij}}=\frac{y_{ij}}{m_{ij}+e_{ij}}\neq \frac{y_{ij}}{m_{ij}}.$ The intrinsic stochastic component operates at the individual-animal level and reflects biological and sampling variation in milk secretion, carryover effects, and time-of-day influences relative to this conditional population mean.

Prediction accuracy is defined as

[6] $$\rho =Cor\left(Y_{i},{\hat{Y}}_{i}\right).$$

Assuming that prediction errors are approximately uncorrelated with the true daily yields, we have

[7] $$<!--ERROR-14INMATHTYPECONVERSION.PLEASECORRECTMANUALLY-->\$Var\left({\hat{Y}}_{i}\right)=Var\left(Y_{i}+{\;}_{i}\right)\approx Var\left(Y_{i}\right)+Var\left({\;}_{i}\right),\$$$

[8] $$<!--ERROR-14INMATHTYPECONVERSION.PLEASECORRECTMANUALLY-->\$Cov\left(Y_{i},{\hat{Y}}_{i}\right)=Cov\left(Y_{i},Y_{i}+{\;}_{i}\right)\approx Var\left(Y_{i}\right).\$$$

The assumption is expected to hold when prediction errors are small and happen randomly. Still, deviations from this assumption may occur if systematic prediction bias exists or if the proportional-yield model inadequately describes certain subsets of the data.

Therefore

[9] ρ=Cov(Yi,Y^i)Var(Yi)Var(Y^i)=Var(Yi)Var(Yi)[Var(Yi)+Var(i)]=Var(Yi)Var(Yi)+Var(i)=1/11+Var(i)Var(Yi)1+Var(i)Var(Yi).

Plugging the decomposed components of [5] into [9] gives

[10] ρ=1/11+σM2+σD2Var(Yi)1+σM2+σD2Var(Yi).

This formula shows that $\sigma_{M}^{2}$ and $\sigma_{D}^{2}$ jointly determine the attainable accuracy; both components can be expressed relative to the phenotypic variance $Var\left(Y_{i}\right).$

Let $\sigma_{M}^{2}=0$ (i.e., all model parameters are correctly specified) and let $x=\frac{\sigma_{D}^{2}}{Var\left(Y_{i}\right)}.$ Then, $\rho \leq \frac{1}{\sqrt{1+x}}\approx 1-\frac{x}{2}+\frac{3x^{2}}{8},$ showing that the accuracy of estimated daily yields is reduced from 1 by approximately $-\frac{x}{2}+\frac{3x^{2}}{8}.$

To further elucidate the components governing prediction accuracy, we derive approximate analytical forms for $\sigma_{M}^{2}$ and $\sigma_{D}^{2}$ below. First, define

$$\Delta_{i}=\frac{y_{ij}}{{\hat{m}}_{ij}}-\frac{y_{ij}}{m_{ij}}=y_{ij}\left(\frac{1}{{\hat{m}}_{ij}}\frac{1}{m_{ij}}\right)=y_{ij}\frac{m_{ij}-{\hat{m}}_{ij}}{{\hat{m}}_{ij}m_{ij}}.$$

Using $Var\left(Z\right)=E\left(Z^{2}\right)-{\left[E\left(Z\right)\right]}^{2},$ the exact variance is obtained as

[11] $$\begin{array}{c} Var\left(\Delta_{i}\right)=Var\left[y_{ij}\frac{m_{ij}-{\hat{m}}_{ij}}{{\hat{m}}_{ij}m_{ij}}\right]=E\left[y_{ij}^{2}\frac{(m_{ij}{\hat{m}}_{ij}){\;}^{2}\;}{{\hat{m}}_{ij}^{2}m_{ij}^{2}}\right]\\ -{\left\{E\left[y_{ij}\frac{m_{ij}-{\hat{m}}_{ij}}{{\hat{m}}_{ij}m_{ij}}\right]\right\}}^{2}. \end{array}$$

Because $m_{ij}-{\hat{m}}_{ij}=\left(\alpha_{j}-{\hat{\alpha }}_{j}\right)+\left(t_{ij}-8\right)\left(\beta -\hat{\beta }\right),$ an equivalent form is

[12] $$\begin{array}{c} Var\left(\Delta_{i}\right)=E\left\{y_{ij}^{2}\frac{{\left[\left({\hat{\alpha }}_{j}-\alpha_{j}\right)+\left(t_{ij}-8\right)\left(\hat{\beta }-\beta \right)\right]}^{2}}{{\hat{m}}_{ij}^{2}m_{ij}^{2}}\right\}\\ -{\left\{E\left[y_{ij}\frac{\left({\hat{\alpha }}_{j}-\alpha_{j}\right)+\left(t_{ij}-8\right)\left(\hat{\beta }-\beta \right)}{{\hat{m}}_{ij}m_{ij}}\right]\right\}}^{2}. \end{array}$$

Under the “perfect-parameter” scenario, where ${\hat{\alpha }}_{j}=\alpha_{j}$ and $\hat{\beta }=\beta ,$ we have $Var\left(\Delta_{i}\right)=0.$ Otherwise, the model parameter-induced error persists.

Because expression [11] depends nonlinearly on the estimated denominator and is, therefore, analytically intractable, we apply a first-order Taylor approximation. Let $\delta_{ij}={\hat{m}}_{ij}-m_{ij}.$ A first-order Taylor expansion of yij/yijm^ijm^ij around *m_ij_* gives

$$\frac{y_{ij}}{{\hat{m}}_{ij}}\approx \frac{y_{ij}}{m_{ij}}+\left({\hat{m}}_{ij}-m_{ij}\right)\left(-\frac{y_{ij}}{m_{ij}^{2}}\right).$$

Subtracting *y_ij_*/*m_ij_* from both sides yields

$$\Delta_{i}=\frac{y_{ij}}{{\hat{m}}_{ij}}-\frac{y_{ij}}{m_{ij}}\approx -\frac{y_{ij}}{m_{ij}^{2}}\left({\hat{m}}_{ij}-m_{ij}\right).$$

Assuming the estimators of yield correction factors are known, we obtain

[13] $$\sigma_{M}^{2}=Var\left(\Delta_{i}\right)\approx E\left(\frac{y_{ij}^{2}}{m_{ij}^{4}}\right)\times Var\left({\hat{m}}_{ij}-m_{ij}\right),$$

where $Var\left({\hat{m}}_{ij}-m_{ij}\right)$ ≈ $Var\left({\hat{\alpha }}_{j}\right)$ + $\text{\textbackslash{}[}(t_{ij}-8){\;}^{2}Var\left(\hat{\beta }\right)\text{\textbackslash{}]}$ + $2\left(t_{ij}-8\right)Cov\left({\hat{\alpha }}_{j},\hat{\beta }\right).$

For the intrinsic stochastic prediction error, we have

$$\frac{y_{ij}}{m_{ij}}-Y_{i}=\frac{y_{ij}}{m_{ij}}-\frac{y_{ij}}{m_{ij}+e_{ij}}=y_{ij}\left(\frac{1}{m_{ij}}-\frac{1}{m_{ij}+e_{ij}}\right).$$

Using the first-order approximation, valid when |*e_ij_*| << *m_ij_*, $\frac{1}{m_{ij}+e_{ij}}\approx \frac{1}{m_{ij}}-\frac{e_{ij}}{m_{ij}^{2}},$ we obtain

[14] $$\frac{y_{ij}}{m_{ij}}-Y_{i}\approx \frac{y_{ij}e_{ij}}{m_{ij}^{2}}.$$

Therefore, the intrinsic stochastic prediction error variance is approximated by

[15] $$\sigma_{D}^{2}\approx Var\left(\frac{y_{ij}e_{ij}}{m_{ij}^{2}}\right).$$

If *y_ij_* and *m_ij_* are treated as known, conditional on them, we obtain

[16] $$\sigma_{D}^{2}\approx E\left(\frac{y_{ij}^{2}}{m_{ij}^{4}}\right)\sigma_{e}^{2},$$

where $Var\left(e_{ij}\right)=\sigma_{e}^{2}$ is the residual variance under the proportional-yield model.

Denote E(yij2mij4)=E{[(yijmij)/(yijmij)mijmij]2}, which represents the expected squared sensitivity of the reconstructed daily yield (*y_ij_*/*m_ij_*) to perturbations in proportional yield (*m_ij_*). It acts as an amplification factor that converts the observed proportional-yield residual variance $\left(\sigma_{e}^{2}\right)$ into the projected daily yield residual variance $\left(\sigma_{D}^{2}\right),$ depending jointly on the herd production level and milking interval structure.

Substituting the variance decomposition of prediction errors into [10] gives

[17] ρ=1/11+E(yij2mij4)[σe2+Var(m^ij−mij)]Var(Yi)1+E(yij2mij4)[σe2+Var(m^ij−mij)]Var(Yi).

Thus, the accuracy of the estimated daily yield is jointly determined by the intrinsic stochastic variance and the parameter estimation error variance; both are amplified by a common factor $E\left(\frac{y_{ij}^{2}}{m_{ij}^{4}}\right)$ and expressed relative to the true daily yield variance $Var\left(Y_{i}\right).$

We demonstrate how this analytical method works using data from four 3× milking Holstein herds (A, B, C, and D) located in 3 US states, with milking data collected between 2023 and 2025. Milking events were classified into 3 categories based on starting time: (1) 0200 to 0959 h, (2) 1000 to 1759 h, and (3) 1800 to 0159 h. Milkings were recorded weekly for each cow up to 120 DIM, followed by monthly testing until 305 DIM or the end of lactation. Data cleaning removed duplicate, missing, and incomplete records, resulting in 15,702 × 3 (herd A), 13,574 × 3 (herd B), 8,515 × 3 (herd C), and 10,821 × 3 (herd D) valid milking observations.

Model parameters were estimated from 3 of the 4 herds (training set) and then used to estimate daily yield in the remaining herd, which served as the independent validation set. This process was repeated in 4 rotations, so that each herd served as the independent validation set exactly once. Prediction accuracy was evaluated using both the analytical approximation formulas and the empirical correlation between observed and estimated daily yields. This cross-validation approach does not explicitly separate the effects of herd-specific management factors, such as milking schedules, equipment calibration, or other operational differences. Training and validating within a single herd is certainly possible, which may yield slightly different accuracy, and it is not used in the present study. In practice, by pooling data from a sufficiently large number of herds, herd-specific effects are expected to be reduced through averaging, resulting in parameter estimates that are well representative of the target population.

Mean estimates of the model parameters from the training and testing sets, respectively, across the replicates of independent validation, are presented in Table 1. Across all 4 herds and for all 3 traits, the estimated intercepts are remarkably stable in the training sets, ranging from approximately 0.331 to 0.336 for milk and protein, and from 0.323 to 0.340 for fat. Under the reparameterized proportional-yield model, these intercepts suggest that each of the 3 milkings contributes roughly one-third of the daily yield. The small deviations among α_1_, α_2_, and α_3_ reflect slight asymmetries among the 3 milking periods but remain minor in magnitude.

**Table 1:** Mean estimates of the section-specific intercepts ${\hat{\alpha }}_{1}$, ${\hat{\alpha }}_{2}$, and ${\hat{\alpha }}_{3}$ and the common linear regression coefficients $\hat{\beta }$ obtained in the training and testing sets^1^

Farm	Training set				Testing set			
	${\hat{\alpha }}_{1}$	${\hat{\alpha }}_{2}$	${\hat{\alpha }}_{3}$	$\hat{\beta }$	${\hat{\alpha }}_{1}$	${\hat{\alpha }}_{2}$	${\hat{\alpha }}_{3}$	$\hat{\beta }$
Milk								
A	0.334	0.331	0.336	0.0367	0.334	0.336	0.330	0.0395
B	0.336	0.332	0.333	0.0373	0.330	0.334	0.336	0.0406
C	0.334	0.332	0.334	0.0379	0.334	0.334	0.335	0.0406
D	0.333	0.335	0.333	0.0402	0.336	0.329	0.335	0.0337
Fat								
A	0.335	0.335	0.330	0.0258	0.336	0.347	0.316	0.0260
B	0.336	0.340	0.323	0.0244	0.334	0.335	0.332	0.0313
C	0.336	0.339	0.326	0.0238	0.335	0.337	0.325	0.0273
D	0.336	0.340	0.324	0.0255	0.334	0.336	0.333	0.0165
Protein								
A	0.332	0.333	0.336	0.0380	0.334	0.336	0.330	0.0391
B	0.334	0.333	0.332	0.0380	0.328	0.336	0.336	0.0425
C	0.332	0.334	0.333	0.0388	0.332	0.335	0.335	0.0412
D	0.331	0.336	0.333	0.0410	0.334	0.331	0.335	0.0344

1 Parameter values from testing sets are listed for comparison only; daily yields for testing animals are estimated using parameter values from the training set.

The intercept estimates from the testing set roughly match those from the training set, with almost negligible differences relative to the scale of proportional yields. This agreement demonstrates that, across herds within the same dairy breed, the proportional-yield structure is highly reproducible and that the training data were representative of the validation herds, despite being collected in 2 of the 5 US regions.

The regression coefficient *β* quantifies the sensitivity of proportional yield to deviations in milking interval from the 8-h baseline. For milk and protein, $\hat{\beta }$ approximately ranges from 0.037 to 0.040 (milk) and from 0.038 to 0.041 (protein) in the training sets. Estimates from the testing sets are slightly more variable (0.034–0.041 for milk and 0.034–0.043 for protein) due to smaller sample sizes, but remain comparable in magnitude. In contrast, $\hat{\beta }$ values are smaller for fat: approximately 0.024 to 0.026 in the training sets and 0.017 to 0.031 in the testing sets. Compared with milk and protein, fat exhibits a smaller linear response to interval length and greater biological variability in fat secretion dynamics. Nonetheless, for a specific trait, $\hat{\beta }$ estimates from training and testing sets remain of the same order of magnitude, indicating that the linear approximation is structurally stable across herds.

The SE are extremely small, indicating highly precise parameter estimates. The SE varied between 0.0002 and 0.0003 for the intercepts and between 0.0002 and 0.0005 for the slope in the training sets, and between 0.0003 and 0.0008 for the intercepts and between 0.0003 and 0.0012 for the slope in the testing sets. This level of precision is consistent with the large number of milking observations per herd. Relatively, the precision is high in the training sets than the testing sets due to significantly more observations.

Variance components and accuracy measures derived from the analytical approach and empirical correlations for daily milk, fat, and protein yields across the 4 herds are listed in Table 2. Several results warrant discussion. First, a notable inflation in variance is observed for the estimated daily yields. For example, the variance of observed daily milk yields, denoted by Var(*Y*), ranges from 78.7 (herd A) to 122.2 (herd C). In contrast, the variance of the estimated daily yield, denoted $Var\left(\hat{Y}\right),$ is consistently larger than Var(*Y*), ranging from approximately 94.8 to 149. Similar patterns are observed for daily fat and protein yields.

**Table 2:** Variance components and accuracy measures derived from the approximation approach, in comparison to estimates obtained through independent validation^1^, ^2^

Farm	Var(*Y*)	$Var\left(\hat{Y}\right)$	<!−−ERROR−14INMATHTYPECONVERSION.PLEASECORRECTMANUALLY−−>$Var()$	$\sigma_{D}^{2}$	$\sigma_{M}^{2}$	ρ_ϵ_	$\rho_{MD}$	$Cor\left(Y,\hat{Y}\right)$
Milk								
A	78.70	94.83	18.05	18.05	0.336	0.902	0.900	0.900
B	98.18	108.7	11.93	11.93	0.261	0.944	0.943	0.944
C	122.2	149.0	33.80	33.79	0.0293	0.885	0.885	0.880
D	113.9	138.3	30.03	30.03	0.324	0.890	0.889	0.885
Fat								
A	0.115	0.175	0.0642	0.0619	3.5E-03	0.801	0.798	0.796
B	0.130	0.172	0.0448	0.0423	1.1E-03	0.862	0.866	0.860
C	0.206	0.281	0.105	0.103	1.9E-04	0.814	0.816	0.793
D	0.177	0.253	0.091	0.0887	1.1E-03	0.813	0.815	0.801
Protein								
A	0.0526	0.0673	0.0162	0.0148	2.8E-04	0.874	0.882	0.872
B	0.0683	0.0790	0.0117	0.0103	3.5E-04	0.924	0.930	0.923
C	0.0997	0.126	0.0344	0.0333	1.6E-05	0.862	0.866	0.853
D	0.0869	0.111	0.0293	0.0283	2.6E-04	0.865	0.868	0.858

1 Var(*Y*) = daily yield phenotype variance; $Var\left(\hat{Y}\right)$ = variance of estimated daily yield; <!−−ERROR−14INMATHTYPECONVERSION.PLEASECORRECTMANUALLY−−>$Var()$ = total prediction error variance; $\sigma_{M}^{2}$ = model parameter-induced prediction error variance; $\sigma_{D}^{2}$ = intrinsic stochastic prediction error variance.

2 $\rho_{\epsilon }=\frac{1}{\sqrt{1+\frac{\mathrm{Var}(\epsilon )}{\mathrm{Var}(Y)}}}$ , $\rho_{\text{MD}}=\frac{1}{\sqrt{1+\frac{\sigma_{M}^{2}+\sigma_{D}^{2}}{\mathrm{Var}(Y_{i})}}}$ and $\mathrm{Cor}(Y,\hat{Y})$ = correlation between the observed and estimated daily yields.

Second, the analytically derived accuracy estimates closely match the correlations between actual and estimated daily yields across the 3 traits. For daily milk yield, the accuracy estimates (*ρ*) range from 0.880 to 0.944. Herd B exhibits the highest accuracy (0.943–0.944) because its prediction error variance is the smallest relative to its total phenotypic variance. Protein yield shows intermediate behavior, with accuracies ranging from 0.853 to 0.930. The analytical approximation closely matches validation results across herds. Herd B again exhibits the highest protein accuracy (0.923–0.930), consistent with the smallest intrinsic variance. Fat yield accuracies are the lowest among the 3 traits, ranging from 0.793 to 0.866, with herd B exhibiting the highest accuracy (0.860–0.866). The lower accuracy for fat compared with milk likely reflects greater biological and sampling variability in fat secretion patterns and a weaker linear relationship between interval length and proportional yield.

For all 3 traits, analytical accuracies closely match empirical correlations. The accuracy hierarchy is milk > protein > fat, likely reflecting differences in biological variability and secretion dynamics. Fat yield appears more sensitive to short-term fluctuations and irregular milking intervals, resulting in a higher intrinsic prediction error.

Third, the intrinsic stochastic variance component dominates the total prediction error variance. For daily milk yield, for example, the intrinsic stochastic variance ranged from 11.9 to 33.8, whereas the model parameter-induced error variance ranged from 0.029 to 0.336. Relatively, the intrinsic stochastic variance constitutes more than 99% of the total prediction error variance. In contrast, the contribution of the model parameter-induced error variance is negligible (≤1%). Similar patterns are observed for fat and protein.

Hence, meaningful improvements in prediction accuracy will require further refinement of the statistical model to effectively reduce the intrinsic stochastic variance and thereby enhance prediction accuracy. For example, including DIM as an additional predictor in the proportional-yield model [1] in a joint analysis of all 4 herds reduced the intrinsic prediction error variance by 0.24% (milk), 9.11% (fat), and 8.88% (protein). Correspondingly, the estimated prediction accuracy increased by 0.51% (milk), 1.67% (fat), and 1.63% (protein). Nevertheless, these improvements remain modest.

An alternative sampling practice is to use the average of multiple-day records. Let ${\bar{y}}_{ij}=\frac{1}{K}\sum_{k=1}^{K}y_{ijk},$ ${\bar{t}}_{ij}=\frac{1}{K}\sum_{k=1}^{K}t_{ijk},$ and ${\bar{m}}_{ij}=\frac{y_{ij}}{t_{ij}}$ represent the pooled records over *K* milking days. Then, the correlation between actual and estimated daily yield is approximated by

[18] $$\rho =\frac{1}{\sqrt{1+\frac{\frac{\sigma_{D}^{2}}{K}+\sigma_{M}^{2}}{Var\left(Y_{i}\right)}}}.$$

Hence, [10] is a special case (*K* = 1) of [18].

The figure in the Graphical Abstract (left bottom panel) shows that accuracy (*ρ*), based on a joint analysis across all 4 herds, increases monotonically as the number of days averaged (*K*) increases from 1 to 7 for all 3 traits, due to a reduction in the intrinsic stochastic variance by a factor of 1/*K*. The largest gains occur between 1 and 3 d, after which the curves gradually plateau, indicating diminishing marginal returns.

Relatively, milk shows the highest accuracy because its intrinsic prediction error variance, $\sigma_{D}^{2},$ is relatively small. Fat shows the lowest accuracy due to a larger intrinsic stochastic variance component, and protein remains intermediate throughout. By about 4 to 5 averaged days, accuracy already exceeds approximately 0.96 to 0.97 for all 3 traits, and additional days provide only small incremental gains. In practice, assessing multiple-day samplings can be considered for milk yields, but it poses practical challenges for fat and protein yields because multiday sampling and testing of fat and protein are more labor-intensive and costly than milk yield recording.

Several limitations of the present study should be acknowledged. First, the analyses were based on 4 US Holstein herds, and the generalizability of the proposed framework to other breeds, production systems, or countries remains to be evaluated. Second, the proportional-yield model assumes a linear relationship with milking interval and, therefore, may not fully capture more complex biological dynamics, particularly for fat secretion. Third, although cross-validation across 4 independent herds provides an assessment of generalizability, it is subject to the limited number of herds. Finally, the analytical derivation relies on the approximation that prediction errors are uncorrelated with the true daily yields, a commonly taken assumption for productive models that may not hold exactly under all conditions.

In conclusion, this study developed and validated an analytical framework to elucidate the determinants of prediction accuracy in estimating daily yields from 3× milking data and to identify avenues for improvement. We demonstrated that intrinsic biological variation in proportional yields is the primary constraint on attainable accuracy, whereas parameter estimation-induced variance is negligible. These findings further indicate that enlarging the training dataset or updating model parameters yields only marginal gains once regression coefficients are estimated with high precision; meaningful improvements must instead arise from reducing intrinsic variability through refinement of the statistical model or enhanced sampling strategies.
